# 

**Published:** 1891-05

**Authors:** 


### CONTENTS

VOL. 38. No. 5.

| | PAGE. |
|---|---|
| La Grippe | 97 |
| The Process of Digestion | 98 |
| Bathing a Sanitary Need | 101 |
| Every Body Likes Her | 103 |
| Laughter | 103 |
| Necessity of Natural Sleep | 105 |
| Over Eating | 106 |
| Capacity for Food of Infants | 106 |
| Giving Animal Food to Infants | 107 |
| Sunny Rooms for Health | 108 |
| Dressing for the Open Air | 108 |
| Bronchitis | 108 |
| Inflamed Eyelids | 109 |
| The Human Figure | 110 |
| Two Remarkable Spiders | 111 |
| Bright's Disease | 112 |
| Monotony in Farm Life | 114 |
| Every Man his own Tailor | 115 |
| Miscellaneous | 116 |
| Literary | 118 |
| The Poet | 120 |

INDEX TO ADVERTISEMENTS OF HALF A PAGE AND OVER.

| | PAGE. |
|---|---|
| Horsford's Acid Phosphate | I |
| Lydia E. Pinkham Med. Co. | II |
| Hall's Journal Premiums | IV |
| Stonington Line | V |
| Ruckel & Hendel | VII |
| Ayer's Cathartic Pills | VII |
| Aerated Oxygen Comp'nd Co. | X |
| Herba Vita | XI |
| J. H. Bunnell & Co | XII |
| Turf, Field & Farm Ass'n | XIII |
| Royal Baking Powder | XV |
| I. S. Johnson & Co. | XV |

